# Complete Genome Sequence of *Vibrio vulnificus* 93U204, a Bacterium Isolated from Diseased Tilapia in Taiwan

**DOI:** 10.1128/genomeA.01005-14

**Published:** 2014-10-02

**Authors:** Wen-Sui Lo, Hwajiun Chen, Chun-Yao Chen, Chih-Horng Kuo

**Affiliations:** a Institute of Plant and Microbial Biology, Academia Sinica, Taipei, Taiwan; b Molecular and Biological Agricultural Sciences Program, Taiwan International Graduate Program, National Chung Hsing University and Academia Sinica, Taipei, Taiwan; c Graduate Institute of Biotechnology, National Chung Hsing University, Taichung, Taiwan; d Department of Life Science, Tzu-Chi University, Hualien, Taiwan; e Institute of Medical Sciences, Tzu-Chi University, Hualien, Taiwan; f Biotechnology Center, National Chung Hsing University, Taichung, Taiwan

## Abstract

*Vibrio vulnificus* 93U204 is a bacterium isolated from a moribund tilapia collected in Kaohsiung, Taiwan. Here, we report the complete genome sequence of this bacterium to facilitate the investigation of its pathogenicity and for comparative analyses with human-pathogenic strains within the same species.

## GENOME ANNOUNCEMENT

*Vibrio vulnificus* is a Gram-negative bacterium commonly found in coastal marine environments throughout the world. While biotype 1 strains within this species are opportunistic pathogens of humans by infection through wounds or contaminated seafood (1), biotype 2 strains are pathogens of eel (2) and tilapia (3) in aquaculture. The biotype 1 strain *V. vulnificus* 93U204 was isolated from a moribund tilapia collected in Kaohsiung, Taiwan (4). To facilitate future characterization of this strain, we determined its complete genome sequence.

The procedure for genome sequencing, assembly, and annotation is based on that described in our previous studies (5–8). Briefly, the Illumina MiSeq platform was used to generate 251-bp reads from one paired-end library (~315-bp insert, 6,818,142 reads), and the Illumina HiSeq 2000 platform was used to generate 126-bp reads from one mate-pair library (~4.1-kb insert, 6,962,414 reads). The *de novo* assembly was performed using AllPaths-LG release 42781 (9). The initial assembly was iteratively improved by mapping the raw reads to the contigs using Burrows-Wheeler Aligner (BWA) version 0.6.2 (10), programmatically checked using the mpileup program in SAMTools package version 0.1.18 (11), and visually inspected using Integrative Genomics Viewer (IGV) version 2.1.24 (12). All gaps were filled by using reads overhanging at the contig margins, and the scaffolding across repetitive regions were confirmed by PCR. The programs RNAmmer (13), tRNAscan-SE (14), and Prodigal (15) were used for gene prediction. For each protein-coding gene, the gene name and product description were initially annotated based on the homologous genes in *V. vulnificus* YJ016 (16), as identified by OrthoMCL (17). Subsequently, BLASTp (18) searches against the NCBI nonredundant (nr) protein database (19) and the Kyoto Encyclopedia of Genes and Genomes (KEGG) database (20) were used to assist manual curation of the annotation.

The complete genome of *V. vulnificus* 93U204 contains three replicons, including two circular chromosomes (chromosome I: 3,315,989 bp, 46.5% G+C content; chromosome II: 1,805,637 bp, 47.1% G+C content) and a circular plasmid p93U204 (5,719 bp, 40.3% G+C content). The first version of annotation includes eight sets of 16S-23S-5S rRNA genes (seven on chromosome I and one on chromosome II), 107 tRNA genes (covering all 20 amino acids), and 4,512 protein-coding genes.

### Nucleotide sequence accession numbers.

The complete genome sequences of *V. vulnificus* 93U204 have been deposited at DDBJ/EMBL/GenBank under the accession numbers CP009261 to CP009263.

## References

[1] Jones MK Oliver JD 2009 *Vibrio vulnificus*: disease and pathogenesis. Infect. Immun. 77:1723–1733. 10.1128/IAI.01046-08 19255188 PMC2681776

[2] Tison DL Nishibuchi M Greenwood JD Seidler RJ 1982 *Vibrio vulnificus* biogroup 2: new biogroup pathogenic for eels. Appl. Environ. Microbiol. 44:640–646 7138004 10.1128/aem.44.3.640-646.1982 PMC242070

[3] Mahmud ZH Wright AC Mandal SC Dai J Jones MK Hasan M Rashid MH Islam MS Johnson JA Gulig PA Morris JG Ali A 2010 Genetic characterization of *Vibrio vulnificus* strains from tilapia aquaculture in Bangladesh. Appl. Environ. Microbiol. 76:4890–4895. 10.1128/AEM.00636-10 20495047 PMC2901738

[4] Chen H Chen C-Y 2014 Starvation induces phenotypic diversification and convergent evolution in *Vibrio vulnificus*. PLoS One 9:e88658. 10.1371/journal.pone.0088658 24551129 PMC3923799

[5] Ku C Lo WS Chen LL Kuo CH 2013 Complete genomes of two dipteran-associated spiroplasmas provided insights into the origin, dynamics, and impacts of viral invasion in *Spiroplasma*. Genome Biol. Evol. 5:1151–1164. 10.1093/gbe/evt084 23711669 PMC3698928

[6] Lo WS Ku C Chen LL Chang TH Kuo CH 2013 Comparison of metabolic capacities and inference of gene content evolution in mosquito-associated *Spiroplasma diminutum* and *S. taiwanense*. Genome Biol. Evol. 5:1512–1523. 10.1093/gbe/evt108 23873917 PMC3762197

[7] Chang TH Lo WS Ku C Chen LL Kuo CH 2014 Molecular evolution of the substrate utilization strategies and putative virulence factors in mosquito-associated *Spiroplasma* species. Genome Biol. Evol. 6:500–509. 10.1093/gbe/evu033 24534435 PMC3971584

[8] Ku C Lo WS Chen LL Kuo CH 2014 Complete genome sequence of *Spiroplasma apis* B31^T^ (ATCC 33834), a bacterium associated with May disease of honeybees (*Apis mellifera*). Genome Announc. 2(1):e01151-13. 10.1128/genomeA.01151-13 24407648 PMC3886961

[9] Gnerre S MacCallum I Przybylski D Ribeiro FJ Burton JN Walker BJ Sharpe T Hall G Shea TP Sykes S Berlin AM Aird D Costello M Daza R Williams L Nicol R Gnirke A Nusbaum C Lander ES Jaffe DB 2011 High-quality draft assemblies of mammalian genomes from massively parallel sequence data. Proc. Natl. Acad. Sci. U. S. A. 108:1513–1518. 10.1073/pnas.1017351108 21187386 PMC3029755

[10] Li H Durbin R 2009 Fast and accurate short read alignment with Burrows–Wheeler transform. Bioinformatics 25:1754–1760. 10.1093/bioinformatics/btp324 19451168 PMC2705234

[11] Li H Handsaker B Wysoker A Fennell T Ruan J Homer N Marth G Abecasis G Durbin R 1000 Genome Project Data Processing Subgroup 2009 The Sequence Alignment/MAP format and SAMtools. Bioinformatics 25:2078–2079. 10.1093/bioinformatics/btp352 19505943 PMC2723002

[12] Robinson JT Thorvaldsdóttir H Winckler W Guttman M Lander ES Getz G Mesirov JP 2011 Integrative genomics viewer. Nat. Biotechnol. 29:24–26. 10.1038/nbt.1754 21221095 PMC3346182

[13] Lagesen K Hallin P Rødland EA Staerfeldt HH Rognes T Ussery DW 2007 RNAmmer: consistent and rapid annotation of ribosomal RNA genes. Nucleic Acids Res. 35:3100–3108. 10.1093/nar/gkm160 17452365 PMC1888812

[14] Lowe TM Eddy SR 1997 tRNAscan-SE: a program for improved detection of transfer RNA genes in genomic sequence. Nucleic Acids Res. 25:955–964. 10.1093/nar/25.5.0955 9023104 PMC146525

[15] Hyatt D Chen GL LoCascio PF Land ML Larimer FW Hauser LJ 2010 Prodigal: prokaryotic gene recognition and translation initiation site identification. BMC Bioinformatics 11:119. 10.1186/1471-2105-11-119 20211023 PMC2848648

[16] Chen CY Wu KM Chang YC Chang CH Tsai HC Liao TL Liu YM Chen HJ Shen ABT Li JC Su TL Shao CP Lee CT Hor LI Tsai SF 2003 Comparative genome analysis of *Vibrio vulnificus*, a marine pathogen. Genome Res. 13:2577–2587. 10.1101/gr.1295503 14656965 PMC403799

[17] Li L Stoeckert CJ Roos DS 2003 OrthoMCL: identification of ortholog groups for eukaryotic genomes. Genome Res. 13:2178–2189. 10.1101/gr.1224503 12952885 PMC403725

[18] Camacho C Coulouris G Avagyan V Ma N Papadopoulos J Bealer K Madden TL 2009 BLAST+: architecture and applications. BMC Bioinformatics 10:421. 10.1186/1471-2105-10-421 20003500 PMC2803857

[19] Benson DA Karsch-Mizrachi I Clark K Lipman DJ Ostell J Sayers EW 2012 GenBank. Nucleic Acids Res. 40:D48–D53. 10.1093/nar/gkr1202 22144687 PMC3245039

[20] Kanehisa M Goto S 2000 KEGG: Kyoto Encyclopedia of Genes and Genomes. Nucleic Acids Res. 28:27–30 http://nar.oxfordjournals.org/content/28/1/27.long 10592173 10.1093/nar/28.1.27 PMC102409

